# Selection of Wheat (*Triticum aestivum* L.) Genotypes for Salinity Tolerance Based on Yield and Ionic Attributes Under Saline Soil Conditions

**DOI:** 10.3390/life16050788

**Published:** 2026-05-08

**Authors:** Rahma Alshamrani, Soleman M. Al-Otayk, Ibrahim S. Elbasyoni, Mohamad I. Motawei

**Affiliations:** 1 Department of Biological Sciences, Faculty of Science, King Abdulaziz University, Jeddah 21589, Makkah, Saudi Arabia; ralshamrani@kau.edu.sa; 2 Department of Plant Production, College of Agriculture and Food, Qassim University, Buraydah 52571, Qassim, Saudi Arabia; satiek@qu.edu.sa; 3 Plant Science Program, Biological and Environmental Science and Engineering Division, King Abdullah University of Science and Technology (KAUST), Thuwal 23955, Makkah, Saudi Arabia; ibrahim.elbasyoni@kaust.edu.sa; 4 Crop Science Department, Faculty of Agriculture, Damanhour University, Damanhour 22516, Egypt

**Keywords:** wheat, plants, salinity stress, genotypic variation, ionic balance, grain yield, K^+^/Na^+^ ratio

## Abstract

Salinity is a major abiotic stress that limits wheat productivity in arid and semi-arid regions. The present study evaluated 20 wheat (*Triticum aestivum* L.) genotypes, including local and improved varieties, under saline soil conditions (ECe ≈ 6.3 and 12.5 dS m^−1^) to assess their performance and tolerance mechanisms. The experiment was conducted using a randomized complete block design with three replicates. Data were recorded for grain yield, number of spikes per square meter, number of kernels per spike, 1000-grain weight, sodium (Na^+^), potassium (K^+^), and K^+^/Na^+^ ratio. Analysis of variance revealed significant differences among the genotypes for all traits. Grain yield ranged from 0.51 t ha^−1^ to 1.14 t ha^−1^, with Bhan 2000, Local, P7, and Sakha 93 showing superior performance, whereas IC15, P6, and IC96 were most affected. A strong positive correlation was observed between grain yield and both kernels per spike (r = 0.75) and K/Na ratio (r = 0.55), whereas Na content was negatively correlated with yield (r = −0.35). Genotypes with higher K^+^/Na^+^ ratios exhibited better ionic balance and salt tolerance. Based on the combined evaluation of productivity and ionic homeostasis, Bhan 2000, Local, P7, and Sakha 93 were clearly identified as the most salt-tolerant genotypes. These genotypes maintained higher grain yields together with optimal K^+^/Na^+^ ratios, reflecting efficient ionic regulation mechanisms. The integrated approach adopted in this study strengthens selection accuracy and highlights these genotypes as promising candidates for cultivation in saline environments and as donor parents in wheat breeding programs targeting salinity tolerance.

## 1. Introduction

Salinity is one of the most severe environmental stresses affecting agricultural productivity worldwide, particularly in the irrigated regions of arid and semi-arid climates. Globally, more than 800 million hectares of land are salt-affected, and this area continues to expand because of secondary salinization, poor irrigation management, and low rainfall [1,2]. Wheat (*Triticum aestivum* L.), a major staple crop for more than one-third of the global population, is moderately sensitive to salinity, with growth and yield substantially reduced at electrical conductivity (ECe) values exceeding 6 dS m^−1^ [3].

Salinity stress affects wheat plants through osmotic and ionic mechanisms, reducing water uptake, impairing photosynthesis, and causing ion toxicity. The accumulation of sodium (Na^+^) and chloride (Cl^−^) in leaf tissues disrupts metabolic processes, whereas potassium (K^+^) depletion limits enzyme activity and osmotic regulation [4]. Therefore, the K^+^/Na^+^ ratio is widely regarded as a key physiological marker of salinity tolerance, as plants that maintain high K^+^ and low Na^+^ concentrations can sustain growth and productivity under saline conditions [5]. Osmotic adjustment is achieved through the accumulation of compatible solutes such as proline, glycine betaine, and soluble sugars, which help maintain cellular water balance and stabilize proteins and membranes under stress conditions [6,7]. Additionally, enhanced antioxidant activity mitigates reactive oxygen species (ROS)-induced damage, protecting cellular structures and metabolic functions [8]. These physiological traits are closely linked with the regulation of stress-responsive genes, providing valuable targets for breeding programs [9,10].

Genetic variability among wheat genotypes provides an opportunity to identify and select salt-tolerant lines based on their physiological and yield-related traits [11]. Breeding programs have increasingly focused on integrating physiological screening with yield performance under stress to improve the accuracy of selection [12]. However, genotype-dependent responses to salinity often differ across environments, necessitating a multi-trait evaluation to effectively classify tolerant and sensitive genotypes in different environments. In this context, the novelty of the present study lies in its integrated, multi-trait assessment of wheat genotypes under saline conditions, combining ionic regulation (Na^+^, K^+^, and K^+^/Na^+^) with yield performance to improve the reliability of tolerance classification. Therefore, the present study was conducted to evaluate the performance of 20 diverse wheat genotypes under saline soil conditions, assess their ionic regulation capacity through Na^+^, K^+^, and K^+^/Na^+^ measurements, and identify salt-tolerant genotypes that can serve as potential parental lines for wheat improvement under salt-affected soils.

## 2. Materials and Methods

### 2.1. Plant Materials and Experimental Design

The experiment was conducted in 2021 and 2022 in a greenhouse under environmental conditions at the Qassim University Agricultural Research and Experimental Station. The average temperature of the greenhouse during the day was 28.5 °C, while night temperature averaged 18 ± 2 °C. Relative humidity ranged from 45–60%. Ventilation and cooling systems were used to prevent excessive heat accumulation. Wheat genotypes included five genotypes from the International Center for Agricultural Research in the Dry Areas (ICARDA), seven genotypes imported from Pakistan, five Australian genotypes, one American genotype (Yocora Rojo), one Egyptian genotype (Sakha 93), and one local genotype (Sama) (Table 1). Two salinity levels of irrigated water (6.3 and 12.5 dS m^−1^ NaCl) and a control (3.1 dS m^−1^) were applied. Each experimental unit consisted of pots filled with a homogenized soil mixture. The soil was characterized prior to planting: texture (sandy loam), pH (8.1), organic matter (1.3%), and available nutrient values were 28, 12, 35, and 18 mg kg^−1^ for N, P, K, and Mg, respectively.

The experimental design was a split-plot design. The whole plots were salinity treatments, and subplots were wheat genotypes with three replications in each salinity treatment. Randomization of subplots was performed independently within each main plot to minimize positional effects. After trial establishment, plants were clipped once to ensure uniform growth before imposing salinity treatments. Saline irrigation was applied using a controlled irrigation schedule based on maintaining soil moisture near field capacity. The volume of irrigation water was measured for each pot to ensure consistent application, and leaching was minimized to maintain target salinity levels. Standard agronomic practices were followed throughout the growing season.

### 2.2. Measured Traits

The following yield-related parameters were recorded at physiological maturity:

- Number of spikes per square meter (spikes m^−2^);
- Number of kernels per spike;
- Thousand-grain weight (g);
- Grain yield: Grain yield was harvested from all plants within each experimental unit (0.25 m × 0.25 m) and initially recorded as g plot^−1^. All grain yield values presented in results are therefore expressed in g m^−2^ and t ha^−1^.

### 2.3. Determination of K^+^ and Na^+^ Concentrations

For ionic determination, plant samples were collected at the designated growth stage, washed with distilled water, and oven-dried at 70 °C until constant weight. Dried samples were ground to a fine powder. Approximately 0.5 g of ground material was digested using a nitric acid–perchloric acid mixture. Sodium (Na^+^) and potassium (K^+^) concentrations in the digested solutions were determined using a flame photometer (Jenway PFP7, Bibby Scientific, UK), as described by AOAC (1990). Calibration curves were prepared using standard solutions before each batch of analysis, and instrument performance was verified using quality control samples.

### 2.4. Statistical Analysis

Data were analyzed using analysis of variance (ANOVA) appropriate for a split-plot design. Salinity treatments and genotypes were considered fixed effects, and replication was treated as a random effect. Assumptions of normality and homogeneity of variance were checked using residual diagnostics (Shapiro–Wilk and Levene’s tests). When necessary, data were transformed to meet ANOVA assumptions. Mean comparisons were performed using Tukey’s HSD post hoc test at 0.05 Probability. The coefficient of variation (CV) was listed to measure the precision of the experiment. All analyses of variance were computed using the MSTATC microcomputer program (MSTATC, 1990).

Pearson’s correlation coefficients were computed among all traits to determine the strength and direction of linear relationships. A correlation heat-map was constructed using the *Seabon* package in Python v3.10. The color gradient ranged from blue (negative correlation) to red (positive correlation), with the scale bar indicating correlation strength from −1.0 to +1.0.

## 3. Results

### 3.1. Performance of Wheat Genotypes Under Salinity Stress

The combined mean of both seasons revealed that increasing salt concentrations caused a substantial reduction in all yield components and physiological traits of wheat (Table 2). Grain yield declined progressively from 1.61 t ha^−1^ under control conditions to 0.48 t ha^−1^ at 6.3 dS m^−1^ and 0.30 t ha^−1^ at 12.5 dS m^−1^. This represents yield reductions of 70.2% and 81.4%, respectively, compared with the control. Other yield components showed similar decreasing trends. The number of spikes per m^2^ decreased from 17.75 in the control to 13.60 at 12.5 dS m^−1^, while kernels per spike dropped from 13.80 to 6.95, indicating that high salinity impairs spike initiation, pollination, and grain setting. Thousand-grain weight also declined markedly, from 38.40 g in the control to 18.25 g at the highest salinity.

Significant variation was observed among the 20 wheat genotypes for all measured traits under saline conditions (Table 3). Grain yield ranged from 0.51 t ha^−1^ (IC 15) to 1.14 t ha^−1^ (Bhan 2000), indicating a wide genotypic diversity in salinity tolerance. Genotypes Bhan 2000, Local, P7, and Sakha 93 exhibited the highest yields, suggesting greater adaptability and physiological resilience under salt stress. In contrast, IC 15, P6, and IC 96 recorded the lowest yields, likely due to higher ionic toxicity or reduced osmotic adjustment.

### 3.2. Ion Accumulation (Na^+^ and K^+^)

Significant genotypic differences were observed in the accumulation of sodium (Na^+^) and potassium (K) (Table 3). The sodium concentration ranged from 20.75 mg kg^−1^ (Bhan 2000) to 43.65 mg kg^−1^ (Sis 13), whereas potassium ranged between 35.35 and 40.55 mg kg^−1^. The K^+^/Na^+^ ratio, a key indicator of ionic balance and salinity tolerance, varied substantially between genotypes. Higher K^+^/Na^+^ ratios were found in Bhan 2000 (3.01), P7 (2.41), and P2 (2.11), reflecting their efficient Na^+^ exclusion and K retention. Overall, genotypes Bhan 2000, P2, P7, and Local demonstrated higher grain yield, balanced ion homeostasis (high K^+^/Na^+^), and desirable yield components, indicating better adaptability to salinity stress. Conversely, IC15, P6, and P8 were the most affected, showing reduced yields and lower K^+^/Na^+^ ratios.

### 3.3. Correlation Heatmap Among Traits

**Figure 1:**
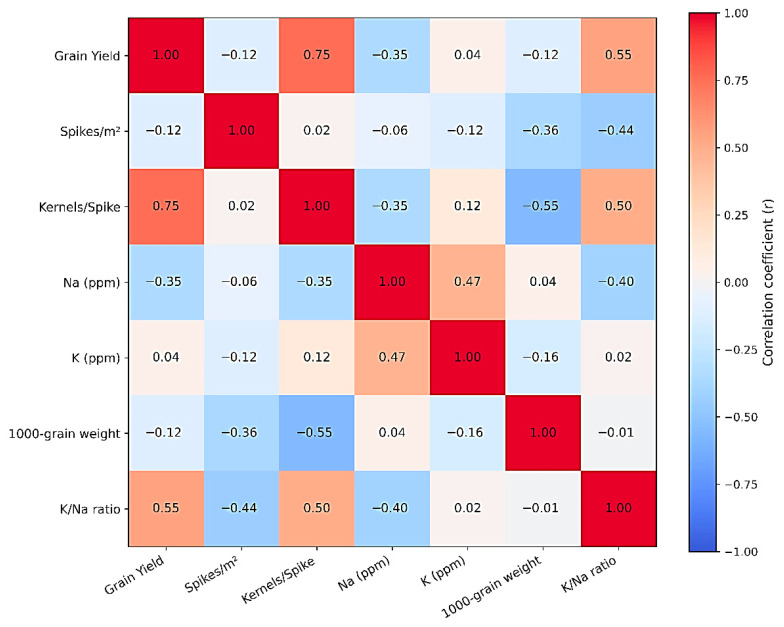
Heatmap of the correlation coefficients among yield, yield components, and ionic traits of wheat genotypes.

The correlation heatmap (Figure 1) revealed clear relationships among grain yield, yield components, and ionic traits of wheat genotypes under salinity stress. Grain yield was strongly positively correlated with the number of kernels per spike (r = 0.75) and the K^+^/Na^+^ ratio (r = 0.55), indicating that genotypes with higher reproductive efficiency and better ionic balance achieve greater yield under saline conditions. Conversely, grain yield showed a moderate negative correlation with Na^+^ content (r = −0.35), suggesting that excessive sodium accumulation reduces yield formation. Sodium concentration was positively correlated with potassium content (r = 0.47), reflecting a partial ionic compensation mechanism under stress. However, Na^+^ exhibited a negative association with the K^+^/Na^+^ ratio (r = −0.40), highlighting that genotypes capable of maintaining a high K^+^/Na^+^ ratio are more salt-tolerant. Overall, these correlations indicate that both reproductive traits (kernels per spike) and ionic homeostasis (high K^+^/Na^+^ ratio) are key determinants of salinity tolerance in wheat, while excessive Na^+^ accumulation is detrimental to yield.

### 3.4. Trait Clustering

**Figure 2:**
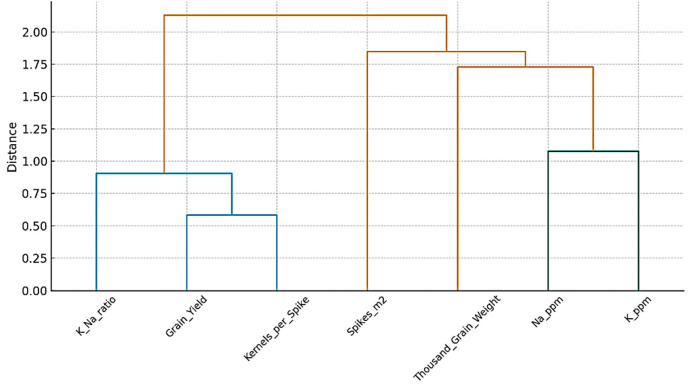
Hierarchical Clustering of Wheat Traits. The color gradient ranged from blue (negative correlation) to red (positive correlation), with the scale bar indicating correlation strength from −1.0 to +1.0.

Hierarchical cluster analysis of yield and ionic traits revealed distinct groupings among the assessed features under salinity stress (Figure 2). Grain yield clustered closely with kernels per spike and the K^+^/Na^+^ ratio, forming a tight sub-cluster at a short distance. This highlights the strong positive relationships among these traits, indicating that higher reproductive efficiency and improved ionic balance are key determinants of enhanced yield under saline conditions. Spikes per m^2^ and thousand-grain weight formed a separate cluster, representing an independent set of yield components that contribute to productivity but are less directly associated with overall grain yield under salinity stress. Ion accumulation traits (Na^+^ and K^+^ content) formed their own distinct cluster, clearly separated from the yield and yield-component clusters. The large clustering distance between Na^+^ content and grain yield emphasize their opposing effects, with excessive Na^+^ negatively impacting productivity. Overall, the dendrogram demonstrates the importance of ionic balance (especially the K^+^/Na^+^ ratio) and kernel number in regulating grain yield in saline environments.

### 3.5. Heatmap of Genotypic Performance

**Figure 3:**
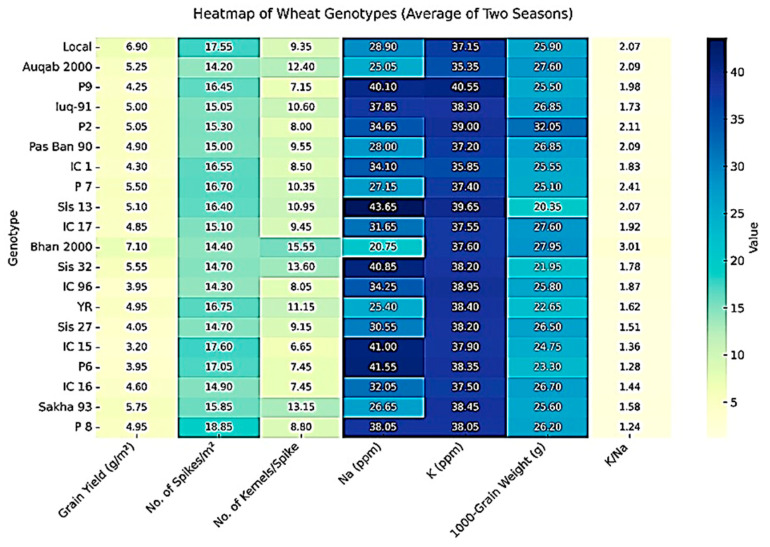
Heatmap of Phenotypic Performance of Wheat Genotypes.

The heatmap (Figure 3) showed significant differences among wheat genotypes for yield components and ion accumulation. Genotypes like Bhan 2000, Local and Sakha 93 were relatively high in grain yield along with spike number coupled with moderate Na^+^ content and favorable K^+^/Na^+^ ratios. On the other hand, some genotypes such as IC 15 and IC 96, and P6 had higher Na accumulation with low grain yield, suggesting more sensitivity in salinity response. The genotypes with the highest K^+^/Na^+^ ratios, namely, Bhan 2000, P7, and P2 tended to perform better in yield attributes, demonstrating the link between ionic balance and tolerance mechanisms. Therefore, the heatmap clearly distinguishes tolerant and sensitive genotypes and demonstrates that improved yield under salinity is closely linked to maintaining a favorable ionic balance.

### 3.6. Cluster Analysis of Wheat Genotypes Under Salinity Stress

**Figure 4:**
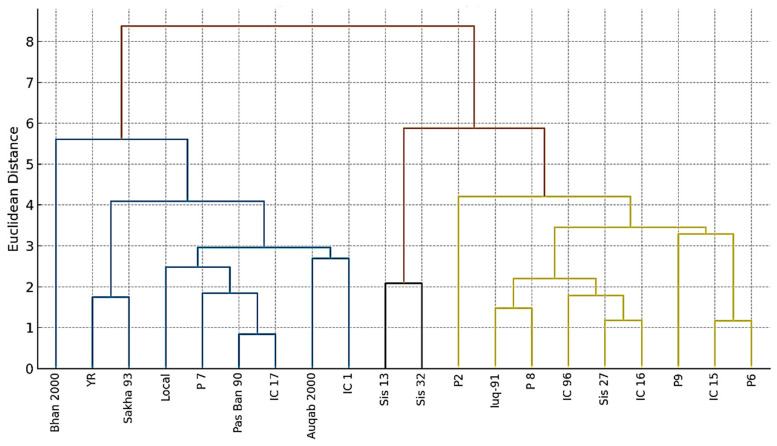
Cluster dendrogram of 20 wheat genotypes based on yield and ionic traits under salinity stress using Ward’s method of hierarchical clustering. Genotypes were grouped into three distinct categories: tolerant (blue), moderately tolerant (orange), and sensitive (yellow).

Wheat genotypes were hierarchically clustered based on yield and ionic characteristics, separating the 20 genotypes into three major groupings (Figure 4). The first cluster comprised salt-tolerant genotypes, including Bhan 2000, Sakha 93, Local, and P7. These genotypes were characterized by higher grain yield, lower Na^+^ accumulation, and higher K^+^/Na^+^ ratios, indicating superior physiological adaptation to saline conditions. The second cluster represented moderately tolerant genotypes, such as Auqab 2000, P2, Pasban-90, IC-17, and Yocora Rojo. These genotypes exhibited intermediate yield performance and moderate ionic balance, suggesting partial tolerance to salinity stress. The third cluster included salt-sensitive genotypes, namely IC 15, P6, P8, Sis-13, and IC 96, which were characterized by low grain yield, elevated Na^+^ accumulation, and reduced K^+^/Na^+^ ratios.

## 4. Discussion

In our research, notable genotypic variations in grain yield, yield components, ion concentrations (Na^+^, K^+^), and K^+^/Na^+^ ratios were evident under saline conditions. Genotypes “Bhan 2000, P2, P7, and Local” demonstrated relatively high yields alongside advantageous K^+^/Na^+^ ratios, while IC15, P6, and P8 showed lower yields and less favorable K^+^/Na^+^ ratios. This trend is consistent with the prevailing understanding that salt tolerance in cereals frequently correlates with effective management of Na^+^ exclusion or compartmentalization, as well as the retention or uptake of K^+^, resulting in a beneficial K^+^/Na^+^ ratio (or reduced Na^+^ toxicity) [6].

The superior performance of tolerant genotypes can be attributed to efficient ion transport and compartmentalization. Membrane transporter families such as HKT, SOS, and NHX regulate Na^+^ exclusion, retrieval from xylem, and vacuolar sequestration [13]. The high K^+^/Na^+^ ratios observed in Bhan 2000 and related genotypes suggest effective operation of these transporters, maintaining cytosolic ion homeostasis and protecting enzyme function. Similar mechanisms have been reported in recent genomic studies, where overexpression of HKT1;5 and NHX1 enhanced salinity tolerance in wheat [6,14]. Genotypes with high Na^+^ and low K^+^/Na^+^ (e.g., P8, P6) likely experienced ionic toxicity and disrupted enzyme or membrane functions, reducing growth and grain filling.

Across all parameters, Bhan 2000 consistently showed the best performance, combining high yield and favorable ionic regulation, followed by Local, P7, and Sakha 93. These genotypes can be classified as salt-tolerant and could serve as parental lines for breeding programs targeting saline environments. In contrast, IC 15, P6, and IC 96 appeared **salt-sensitive**, showing reduced yield and poor ionic homeostasis. The observed variability may stem from genetic differences in ion transport efficiency, osmotic regulation, and antioxidant capacity, as previously reported in other wheat studies [11,15].

The correlation analysis revealed insights into the physiological mechanisms that control the yield performance of wheat genotypes in saline environments. Grain yield was positively associated with the number of kernels per spike and 1000-grain weight, which are major determinants of productivity under saline environments [1,16]. This aligns with the findings of Al-Karaki and Al-Khateeb [17], who indicated that salt-resistant genotypes exhibit enhanced reproductive growth owing to consistent photosynthetic function and decreased spikelet loss.

A significant discovery is the favorable correlation between grain yield and K/Na ratio (r = 0.55), highlighting the role of ionic balance in sustaining metabolic activities during saline stress. An increased K/Na ratio shows effective K^+^ retention and Na^+^ exclusion processes, which maintain enzyme function, osmotic balance, and stomatal control [18]. These findings support earlier studies by [19,20], highlighting that wheat genotypes that can maintain higher K^+^ accumulation compared to Na^+^ demonstrate enhanced salt tolerance and yield stability. Genotypes such as P8 (1.24), P6 (1.28), and IC 15 (1.36) exhibited lower K^+^/Na^+^ ratios, suggesting an ionic imbalance that likely impaired metabolic activity and yield performance. These results align with previous findings that maintaining a higher K^+^/Na^+^ ratio reflects efficient Na^+^ exclusion and sustained K^+^ uptake, which are necessary for maintaining stomatal regulation, ATP synthesis, and metabolic stability under saline conditions [4,12].

The inverse relationship between Na levels and yield (r = −0.35) underscores the harmful effects of sodium toxicity, which compromises cell membrane structure, photosynthetic performance, and nutrient absorption [21]. The moderate positive correlation between Na^+^ and K^+^ (r = 0.47) suggests a partial compensatory uptake mechanism, where tolerant genotypes might manage Na intake to maintain osmotic balance without significantly disrupting K homeostasis.

Interestingly, the 1000-grain weight exhibited a negative correlation with kernels per spike (r = −0.55), indicating a trade-off between grain size and grain number in saline conditions. Comparable results were noted by [22], who found that salinity stress restricts the distribution of assimilates to growing grains, leading to decreased weight of individual grains even though the number of grains per spike increased.

Both heatmap and cluster analyses consistently grouped high-yielding genotypes with favorable ionic traits, confirming that Na^+^ exclusion and K^+^ retention are crucial for performance under salinity [8,11]. From a breeding perspective, the identification of tolerant genotypes such as Bhan 2000, Local, P7, and Sakha 93 provides valuable genetic resources for developing salt-tolerant cultivars. These genotypes can serve as parental lines for conventional breeding or marker-assisted selection targeting key genes related to ion transport, osmotic adjustment, and ROS detoxification [9,23].

The correlation pattern found in this study suggests that the K^+^/Na^+^ ratio and the number of kernels per spike are dependable selection criteria for salt tolerance in wheat. Genotypes that integrate effective ion regulation with consistent reproductive growth are expected to sustain greater yields under saline irrigation. Subsequent breeding initiatives need to emphasize the incorporation of physiological assessments for K^+^/Na^+^ balance alongside yield component analysis to improve selection efficiency.

The key discovery was recognizing Bhan 2000 as a highly salt-tolerant genotype, creating its own unique cluster. Bhan 2000 consistently showcased exceptional resilience, displaying the highest K^+^/Na^+^ ratio of all genotypes. An elevated K^+^/Na^+^ ratio is an essential physiological characteristic for salt tolerance, since potassium is crucial for enzyme activation and osmotic balance, whereas sodium becomes toxic in high amounts. Bhan 2000’s capacity to sustain this advantageous ratio implies effective processes for sodium exclusion or improved potassium retention in its leaves, an essential characteristic of tolerant varieties [5].

The results of the cluster analysis provide a clear roadmap for wheat breeding programs aimed at improving salt tolerance. Bhan 2000 emerges as an excellent candidate for use as a parent in hybridization programs to transfer its strong salt tolerance traits to high-yielding but sensitive genotypes. The moderately tolerant genotypes from cluster analysis could be valuable for breeding programs targeting areas with moderate salinity, or they could be used in gene pyramid strategies to combine different tolerance mechanisms.

## 5. Conclusions

In conclusion, this research verifies the existence of significant genetic variation for salt tolerance in wheat. Cluster analysis effectively classified the 20 genotypes into sensitive, moderately tolerant, and highly tolerant categories. The genotype Bhan 2000 was recognized as an exceptional source of salt tolerance mainly linked to its capacity to sustain a high K^+^/Na^+^ ratio. The findings highlight the significance of combining physiological characteristics such as ionic balance with agronomic success for the efficient selection and breeding of salt-tolerant wheat varieties appropriate for growing in saline-impacted areas. Future studies must aim to confirm these results in real-world scenarios and clarify the molecular and genetic foundations of the tolerance seen in Bhan 2000.

## Figures and Tables

**Table 1:** List of the 20 wheat genotypes used in this study.

No	Genotype Name	Genotype Source	Pedigree/Note (Corrected)
1	Auqab 2000	Pakistan	CROW (sib)/NACOZARI-76//(sib) BOBWHITE
2	Inqalab-91 (Inq-91)	Pakistan	WL-711/CROW “S”
3	Pasban 90	Pakistan	INIA F66/TH.DISTICHUM//INIAF66/3/GENARO T81
4	Sis 13	Pakistan	Internal breeding line.
5	Sis 32	Pakistan	Internal breeding line.
6	Sis 27	Pakistan	Internal breeding line.
7	Bhan 2000	Pakistan	Internal breeding line.
8	P2	Australia	AUS-030852
9	P6	Australia	AUS-030856
10	P7	Australia	AUS-030857
11	P8	Australia	AUS-030858
12	P9	Australia	AUS-030859
13	IC1	ICARDA	Line-1 ICARDA-1 st RDRN0607
14	IC15	ICARDA	Line-15 ICARDA-1 st RDRN0607
15	IC16	ICARDA	Line-16 ICARDA-1 st RDRN0607
16	IC17	ICARDA	Line-17 ICARDA-1 st RDRN0607
17	IC 96	ICARDA	Line-96 ICARDA-1 st RDRN0607
18	Sakha 93	Egypt	SAKHA-92/TR-810328 [3555]
19	* YR	USA	American genotype
20	** Sama	Saudi Arabia	Saudi cultivar release document.

* Yocora Rojo: commercial genotype grows commonly in Saudi Arabia; ** Sama: local genotype.

**Table 2:** Combined analysis (mean of both seasons) for the effect of salt treatments on wheat traits.

Salt Treatment	Grain Yield (g/m^2^)	Grain Yield (t ha^−1^)	Spikes/m^2^	Kernels/Spike	Na^+^ (mg kg^−1^)	K^+^ (mg kg^−1^)	1000-Grain Weight (g)	K^+^/Na^+^
**Control** (3.1dS m^−1^)	161.0 a *	1.61 a	17.7 a	13.8 a	10.45 c	35.15 b	38.4 a	3.13
6.3 dS m^−1^	48.0 b	0.48 b	16.2 b	8.9 b	42.25 b	39.45 a	20.2 b	1.18
12.5 dS m^−1^	30.0 c	0.30 c	13.6	6.9 c	51.60 a	39.85 a	18.25 c	1.07
SD **	±13.9	±0.14	±1.13	±1.18	±3.2	±1.14	±1.32	

* Means followed by different letters under the same column are significantly different at *p* < 0.05. ** Standard deviation (SD).

**Table 3:** Combined analysis (mean of both seasons) for performance of 20 wheat genotypes under saline conditions.

Genotype	Grain Yield (t ha^−1^)	Spikes m^−2^	Kernels Spike^−1^	Na^+^ (mg kg^−1^)	K^+^ (mg kg^−1^)	1000-Grain Weight (g)	K^+^/Na^+^
Bhan 2000	1.14 ^a^*	14.40 ^e^	15.55 ^a^	20.75 ^f^	37.60 ^b^	27.95 ^ab^	3.01 ^a^
Local	1.10 ^a^	17.55 ^ab^	9.35 ^cd^	28.90 ^e^	37.15 ^b^	25.90 ^bc^	2.07 ^b^
Sakha 93	0.92 ^b^	15.85 ^bc^	13.15 ^ab^	26.65 ^ef^	38.45 ^ab^	25.60 ^bc^	1.58 ^c^
P7	0.88 ^bc^	16.70 ^ab^	10.35 ^bc^	27.15 ^ef^	37.40 ^b^	25.10 ^bc^	2.41 ^b^
Sis 32	0.89 ^bc^	14.70 ^cd^	13.60 ^ab^	40.85 ^ab^	38.20 ^ab^	21.95 ^d^	1.78 ^c^
Auqab 2000	0.84 ^cd^	14.20 ^de^	12.40 ^ab^	25.05 ^f^	35.35 ^c^	27.60 ^ab^	2.09 ^b^
Sis 13	0.82 ^cd^	16.40 ^ab^	10.95 ^bc^	43.65 ^a^	39.65 ^a^	20.35 ^d^	2.07 ^b^
P2	0.81 ^cd^	15.30 ^cd^	8.00 ^cd^	34.65 ^cd^	39.00 ^a^	32.05 ^a^	2.11 ^b^
Inq-91	0.80 ^cd^	15.05 ^cd^	10.60 ^bc^	37.85 ^bc^	38.30 ^ab^	26.85 ^ab^	1.73 ^c^
YR	0.79 ^cd^	16.75 ^ab^	11.15 ^bc^	25.40 ^f^	38.40 ^ab^	22.65 ^cd^	1.62 ^c^
Pasban 90	0.78 ^cd^	15.00 ^cd^	9.55 ^cd^	28.00 ^e^	37.20 ^b^	26.85 ^ab^	2.09 ^b^
IC 17	0.78 ^cd^	15.10 ^cd^	9.45 ^cd^	31.65 ^de^	37.55 ^b^	27.60 ^ab^	1.92 ^bc^
IC 16	0.74 ^d^	14.90 ^cd^	7.45 ^de^	32.05 ^de^	37.50 ^b^	26.70 ^ab^	1.44 ^c^
IC 1	0.69 ^de^	16.55 ^ab^	8.50 ^cd^	34.10 ^cd^	35.85 ^c^	25.55 ^bc^	1.83 ^bc^
P9	0.68 ^de^	16.45 ^ab^	7.15 ^de^	40.10 ^ab^	40.55 ^a^	25.50 ^bc^	1.98 ^bc^
Sis 27	0.65 ^ef^	14.70 ^cd^	9.15 ^cd^	30.55 ^de^	38.20 ^ab^	26.50 ^ab^	1.51 ^c^
IC 96	0.63 ^ef^	14.30 ^de^	8.05 ^cd^	34.25 ^cd^	38.95 ^a^	25.80 ^bc^	1.87 ^bc^
P6	0.63 ^ef^	17.05 ^ab^	7.45 ^de^	41.55 ^ab^	38.35 ^ab^	23.30 ^cd^	1.28 ^d^
P8	0.79 ^cd^	18.85 ^a^	8.80 ^cd^	38.05 ^bc^	38.05 ^ab^	26.20 ^bc^	1.24 ^d^
IC 15	0.51 ^f^	17.60 ^ab^	6.65 ^e^	41.00 ^ab^	37.90 ^b^	24.75 ^bc^	1.36 ^d^
SD **	±0.36	±2.91	±3.04	±8.27	±2.95	±3.40	

* Means followed by different letters under the same column are significantly different at *p* < 0.05. ** Standard deviation (SD).

## Data Availability

The original contributions presented in this study are included in the article; further inquiries can be directed at the corresponding author.
